# The pathophysiology of ‘happy’ hypoxemia in COVID-19

**DOI:** 10.1186/s12931-020-01462-5

**Published:** 2020-07-28

**Authors:** Sebastiaan Dhont, Eric Derom, Eva Van Braeckel, Pieter Depuydt, Bart N. Lambrecht

**Affiliations:** 1 Department of Internal Medicine and Paediatrics, Ghent University, Corneel Heymanslaan 10, 9000 Ghent, Belgium; 2 Department of Respiratory Medicine, Ghent University Hospital, Ghent, Belgium; 3 Department of Intensive Care Medicine, Ghent University Hospital, Ghent, Belgium; 4 VIB-UGent Center for Inflammation Research, Ghent, Belgium

**Keywords:** COVID-19, SARS-CoV-2, Respiratory failure, Hypoxemia, Dyspnea, Gas exchange

## Abstract

The novel coronavirus disease 2019 (COVID-19) pandemic is a global crisis, challenging healthcare systems worldwide. Many patients present with a remarkable disconnect in rest between profound hypoxemia yet without proportional signs of respiratory distress (i.e. happy hypoxemia) and rapid deterioration can occur. This particular clinical presentation in COVID-19 patients contrasts with the experience of physicians usually treating critically ill patients in respiratory failure and ensuring timely referral to the intensive care unit can, therefore, be challenging. A thorough understanding of the pathophysiological determinants of respiratory drive and hypoxemia may promote a more complete comprehension of a patient’s clinical presentation and management. Preserved oxygen saturation despite low partial pressure of oxygen in arterial blood samples occur, due to leftward shift of the oxyhemoglobin dissociation curve induced by hypoxemia-driven hyperventilation as well as possible direct viral interactions with hemoglobin. Ventilation-perfusion mismatch, ranging from shunts to alveolar dead space ventilation, is the central hallmark and offers various therapeutic targets.

## Take home message

This review describes the pathophysiological abnormalities in COVID-19 that might explain the disconnect between the severity of hypoxemia and the relatively mild respiratory discomfort reported by the patients.

## Background

In early December 2019, the first cases of a pneumonia of unknown origin were identified in Wuhan, the capital of Hubei province in China. The pathogen responsible for coronavirus disease 2019 (COVID-19) has been identified as a novel member of the enveloped RNA betacoronavirus family and named severe acute respiratory syndrome coronavirus 2 (SARS-CoV-2), due to similarities with SARS-CoV and Middle East Respiratory Syndrome (MERS) viruses. Although much is known about the epidemiology and the clinical characteristics of COVID-19, little is known about its impact on lung pathophysiology. COVID-19 has a wide spectrum of clinical severity, data classifies cases as mild (81%), severe (14%), or critical (5%) [1–3]. Many patients present with pronounced arterial hypoxemia yet without proportional signs of respiratory distress, they not even verbalize a sense of dyspnea [4–8]. This phenomenon is referred as silent or ‘happy’ hypoxemia. Tobin et al. recently presented three cases of happy hypoxemia with P_a_O_2_ ranging between 36 and 45 mmHg in the absence of increased alveolar ventilation (P_a_CO_2_ ranging between 34 and 41 mmHg) [5]. In patients with COVID-19, the severity of hypoxemia is independently associated with in-hospital mortality and can be an important predictor that the patient is at risk of requiring admission to the intensive care unit (ICU) [9, 10]. Since correct recognition of hypoxemia has such an impact on prognosis and timely treatment decisions, we here offer an overview of the pathophysiological abnormalities in COVID-19 that might explain the disconnect between hypoxemia and patient sensation of dyspnea.

## Dyspnea as a sensation

**Fig. 1:**
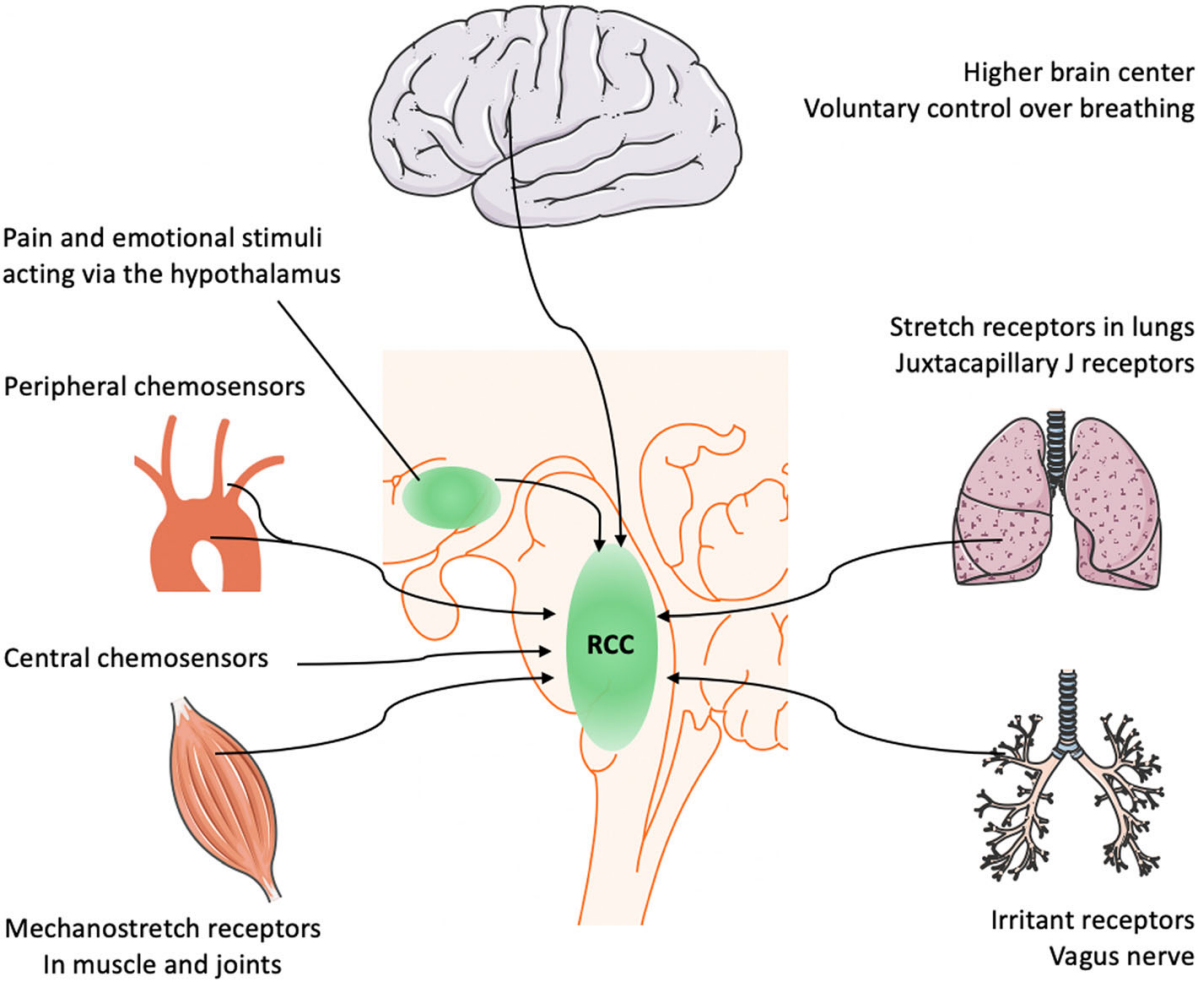
Main inputs affecting respiratory center (RCC)

Breathing is centrally controlled by the respiratory center in the medulla oblongata and pons regions of the brainstem (see Fig. 1) that control the ‘respiratory drive’ to match respiration to the metabolic demands of the body [11, 12]. The main input affecting the respiratory drive is derived from chemical feedback among peripheral and central chemoreceptors. The center is, however, also influenced by higher brain cortex, hypothalamic integrative nociception, feedback from mechanostretch receptors in muscle and lung, and metabolic rate. The output of the respiratory center can be divided into rhythm- (e.g. respiratory rate) and pattern generating (e.g. depth of breathing effort) signals, and these outputs may be controlled independently [11, 13, 14]. Dyspnea is generally defined as a sensation of ‘uncomfortable, difficult, or labored’ breathing and occurs, in general, when the demand for ventilation is out of proportion to the patient’s ability to respond. It should be distinguished from tachypnea (rapid breathing) or hyperpnea (increased ventilation). Dyspnea grading relates to whether this feeling occurs in rest or upon exercise. This semi-quantitative approach of scoring is best exemplified by the frequently used modified Medical Research Council (MRC) dyspnea scale, which categorizes dyspnea from grade 0 (dyspnea only with strenuous exercise) to grade 4 (too dyspneic to leave house or breathless when dressing) in relation to subjects of the same age [15, 16].

Various sensory, pain and emotional stimuli affect the sensation of breathing via the cerebral cortex and hypothalamus [17, 18]. The abnormal sense of muscle effort is another contributor to dyspnea. Conscious awareness of the activation of respiratory muscles is absent in healthy breathing. However, when the respiratory muscles are fatigued or weakened due to altered lung mechanics (e.g. decreased thoracic compliance), breathing may be perceived as a substantial effort [12]. Dyspnea can also be caused by input from the mechanoreceptors in the respiratory tract and the chest wall. Stimulation of vagal irritant receptors (e.g. bronchoconstriction, breathing through an external resistance) appears to intensify dyspnea [12, 19, 20]. The contribution of metabolic rate in modulating sense of dyspnea in critically ill patients remains unclear, despite its well-established role during exercise [11, 21]. The best-known determinants of the respiratory drive are the central and peripheral chemoreceptors. Changes in partial gas pressure of dissolved carbon dioxide in the blood (PaCO_2_) seem the most important component, causing shifts in pH at the level of both the peripheral and central chemoreceptors [11, 12, 22]. At steady state, the arterial PaCO_2_ is determined by the following equation:

$$ PaCO2=\frac{K. VCO2}{Ve.\left(1-\raisebox{1ex}{$ Vd$}\!\left/ \!\raisebox{-1ex}{$ Vt$}\right.\right)}. $$

**Table:** 

K	constant (863 mmHg)
VCO_2_	Rate of CO_2_ production
V_E_	minute ventilation
V_D_	dead space
Vt	tidal volume

The normal response to hypercapnia (caused by increased V_D_, hypoventilation or increased VCO_2_) is an increase in respiratory drive and minute volume ventilation [23]. Hypoxemia itself rather plays a limited role in the sensation of breathlessness experienced by patients with cardiopulmonary disease on the opposite of hypercapnia that creates per se dyspnea [12, 24, 25]. In healthy subjects, the respiratory drive shifts minimally in mild hypoxemia (PaO_2_ 60–65 mmHg), such as resulting from stays at high altitude or experimental hypoxic chambers [11, 26]. Many patients with dyspnea are not hypoxemic, while those who are, usually experience only a slight improvement in symptoms after hypoxemia is corrected with supplemental oxygen therapy [12]. When arterial P_a_O_2_ drops below 40 mmHg, dyspnea often occurs [12]. Of note, the normal response to hypoxemia is a rise in minute ventilation, primarily by increasing tidal volume and respiratory rate. Increased respiratory rate (tachypnea) and tidal volume (hyperpnea) - and not dyspnea - are therefore the most important clinical signs of impending hypoxemic respiratory failure [11, 27]. Furthermore, PaCO_2_ serves as one of the fundamental regulators of cerebral blood flow. Hyperventilation causes decreased PaCO2 which subsequently leads to arterial vasoconstriction thus lowering cerebral blood flow and intracranial pressure. In contrary, increase in PaCO_2_ leads to increased intracranial pressure ultimately leading to deteriorating level of consciousness, altered brainstem reflexes, and altered postural and motor responses [28, 29]. At the bedside, a profound understanding of the pathophysiological determinants of respiratory drive and hypoxemia may promote a more complete comprehension of a COVID-19 of a patient’s clinical presentation and timely management [11].

## Happy hypoxemia in COVID-19

**Fig. 2:**
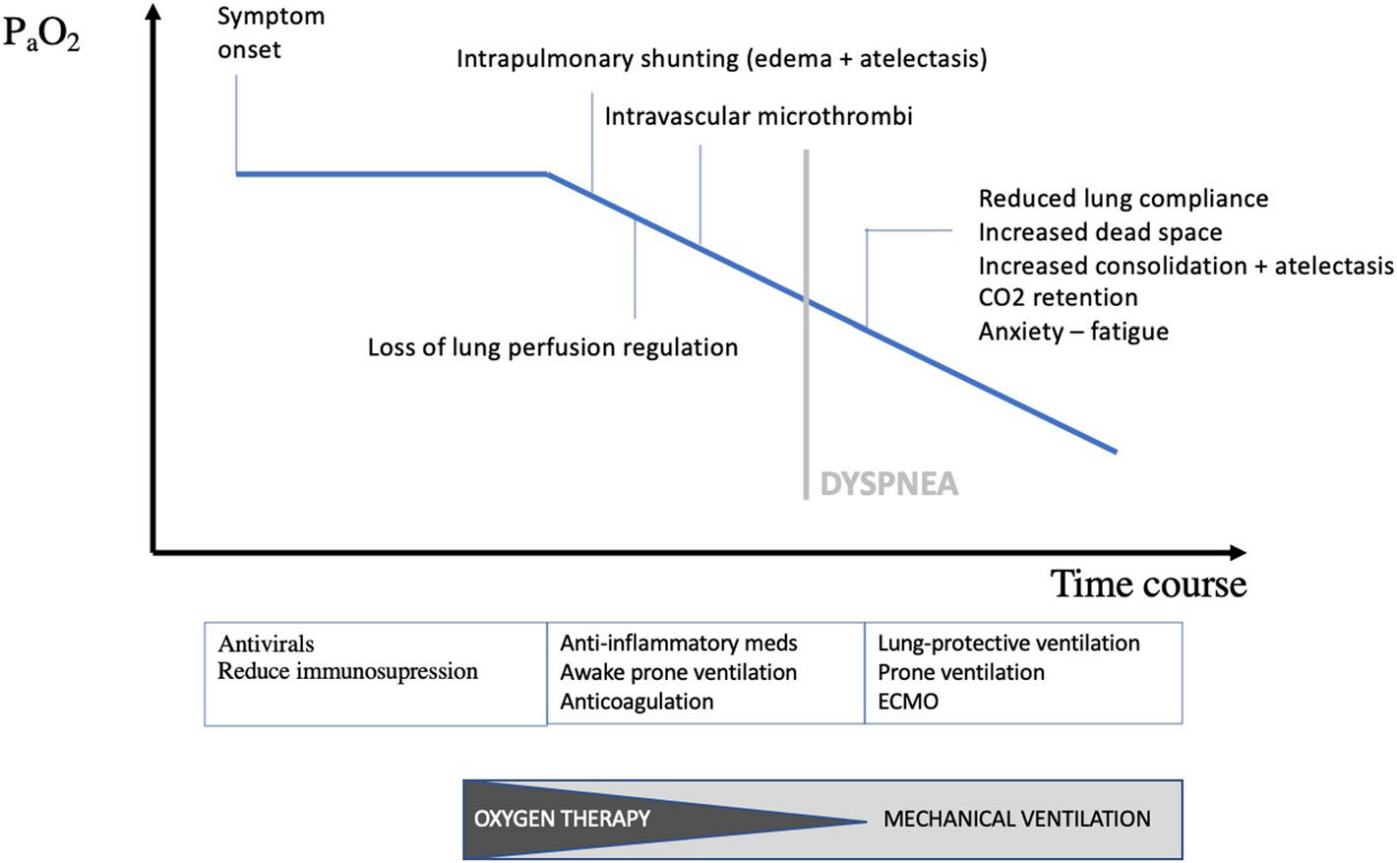
Mechanisms of hypoxemia in COVID-19

The disconnect between the severity of hypoxemia and the relatively mild respiratory discomfort reported by the COVID-19 patients contrasts with the experience of physicians usually treating critically ill patients in respiratory failure [30]. Guan reported dyspnea in only 18.7% of 1099 hospitalized COVID-19 patients, despite low PaO2/FiO2 ratios, abnormal CT scans (86%) and common requirement for supplemental oxygen (41%) [31]. Happy or silent hypoxemia is not exclusively seen in COVID-19, but may also occur in patients with atelectasis, intrapulmonary shunt (i.e. arterio-venous malformations) or right-to-left intracardiac shunt. The adequacy of gas exchange is primarily determined by the balance between pulmonary ventilation and capillary blood flow, referred as ventilation/perfusion (V/Q) matching [32]. In the initial phase of COVID-19, several mechanisms contribute to the development of arterial hypoxemia (see Fig. 2), without a concomitant increase in work of breathing. Rapid clinical deterioration may occur.

### Changes in oxyhemoglobin dissociation curve

Oxygen saturation measured by pulse oximetry (SpO_2_) is often used to detect hypoxemia. However, SpO_2_ should be interpreted with caution in COVID-19. The sigmoid shaped oxyhemoglobin dissociation curve seems to shift to the left, due to induced respiratory alkalosis (drop in PaCO_2_) because of hypoxemia-driven tachypnea and hyperpnea. During hypocapnic periods, the affinity of hemoglobin for oxygen and thus oxygen saturation increases for a given degree of PaO_2_, explaining why SpO_2_ can be well-preserved in the face of a profoundly low PaO_2_ [33–35]. This finding is also seen in high altitude hypoxemia, in which hypocapnia significantly shifts the oxygen-hemoglobin dissociation curve and improves blood oxygen saturation [36]. The alveolar gas equation also predicts that hyperventilation and the resulting drop in the alveolar partial pressure of carbon dioxide leads to an increase in the alveolar partial pressure of oxygen and ultimately lead to an increase in SpO_2_ [22].

There might also be a biological explanation for the leftward shift of the curve in COVID-19. Liu et al. put forward hypotheses about direct viral interaction with the heme group of hemoglobin. According to this theory, heme serum levels are increasing in COVID-19 along with harmful iron ions (Fe^3+^) causing inflammation and cell death (ferroptosis). This leads to the production of large amounts of serum ferritin to bind these free irons in order to reduce tissue damage [37]. In conclusion, SpO_2_ should be interpreted in the light of the presence of hyperventilation (tachypnea, low P_a_CO_2_) and, if possible, P_a_O_2_ via arterial puncture. Measuring the alveolar to arterial oxygen (P(A-a)O_2_ gradient (150 mmHg - PaCO2/0.8 - P_a_O_2_ at sea level) and relating this value to age and supplemental oxygen (age/4 + 4 + 50 (F_i_O_2_–0.21) in mmHg) can be insightful. This can be performed rapidly on a smartphone app [38]. The P(A-a)O_2_ gradient is increased either by V/Q mismatch or by intrapulmonary shunting. Hypoxemia due to V/Q mismatch can be easily corrected by supplemental oxygen therapy whereas pulmonary shunts have a poor response to oxygen therapy [39].

### Causes of hypoxemia in COVID-19

#### Intrapulmonary shunting

Arterial hypoxemia early in SARS-CoV-2 infection is primarily caused by V/Q mismatch and thus persistence of pulmonary arterial blood flow to non-ventilated alveoli, reflected by a marked increase in P(A-a)O_2_ gradient. The infection leads to a modest local interstitial edema, particularly located at the interface between lung structures with different elastic properties, where stress and strain are concentrated [27]. Due to increased lung edema (leading to ground-glass opacities and consolidation on chest imaging), loss of surfactant and superimposed pressure, alveolar collapse ensues and a substantial fraction of the cardiac output is perfusing non-aerated lung tissue, resulting in intrapulmonary shunting [27]. As previously discussed, tidal volume increases during the disease course leading to rising negative inspiratory intrathoracic pressure. The latter, in combination with increased lung permeability due to inflammation, will eventually result in progressive edema, alveolar flooding, and patient self-inflicted lung injury (P-SILI), as first described by Barach in 1938 [11, 40, 41]. Over time, the increased edema will further enhance lung weight, alveolar collapse, and dependent atelectasis, resulting in progressively increasing shunt fraction and further decline of oxygenation which cannot completely be corrected by increasing F_i_O_2_.

#### Loss of lung perfusion regulation

The persistence of high pulmonary blood flow to non-aerated lung alveoli appears to be caused by the relative failure of the hypoxic pulmonary vasoconstriction mechanism (constriction of small intrapulmonary arteries in response to alveolar hypoxia) during SARS-CoV-2 infection, as recently illustrated by Lang et al. using dual-energy CT [42, 43]. Whether the latter mechanism is only triggered by the release of endogenous vasodilator prostaglandins, bradykinin, and cytokines associated with the inflammatory process or also by other yet undefined mechanisms remains to be investigated [33, 44, 45]. Vasoplegia also seems to be influential in the loss of lung perfusion regulation, possibly induced by shear stress on the interfaces between lung structures, as part of the P-SILI spectrum [45–47]. Further, dysregulation of the renin-angiotensin system (RAS) contribute to the pathophysiology of COVID-19 [48–52]. Angiotensin-converting enzyme 2 (ACE2) is the principal functional receptor used by SARS-CoV-2 for cell entry, implying ACE2 internalization [52–54]. ACE2 converts angiotensin II (Ang II) to angiotensin 1–7 (Ang 1–7) and is also important for degrading bradykinin. Hence, diminished levels of ACE2 lead to an increase in Ang II, mediating pulmonary vasoconstriction through agonism at Ang II-receptor, while Ang 1–7 opposes the actions of Ang II [50, 51, 54]. Recently, Liu et al. revealed that serum Ang II levels were linearly associated with viral load and lung injury in COVID-19 [55].

#### Intravascular microthrombi

Endothelial injury is emerging as a central hallmark of COVID-19 pathogenesis, and the cytopathic virus can directly infect lung capillary endothelial cells that express ACE2 [54, 56]. Intravascular microthrombi are the net result of an imbalance between procoagulant and fibrinolytic activity in the presence of acute inflammation and endothelial injury [45, 57–59]. The pro-coagulant activity might result from complement system-mediated activation of clotting, similar to some forms of thrombotic microangiopathy (TMA), or could be due to inhibition of plasminogen activation and fibrinolysis via increased activity of plasminogen activator inhibitor (PAI-1 and -2) which are induced as acute-phase proteins under the influence of IL-6. Diffuse intravascular coagulation (DIC) is also seen in patients with severe COVID-19, mediated via endothelial release of tissue factor and activation of clotting factor VII and XI. Many patients with COVID-19 develop elevated D-dimers suggesting the formation of blood clots. D-dimer levels on admission are used to predict in-hospital mortality in COVID-19, and DIC present much more frequently (71%) in COVID-19 patients with a dismal prognosis, versus only 0,6% of survivors [60–63]. Autopsy of the lungs after severe disease showed fibrin deposition, diffuse alveolar damage, vascular wall thickening, and frequently occurring complement-rich microthrombi occluding lung capillaries and larger thrombi causing pulmonary artery thrombosis and embolism [63–65]. Hypercoagulable state leads to further deterioration in V/Q mismatch and lung tissue damage. Moreover, coagulation is also modulated by activating C-reactive protein and ensuing complement activation and hepatic synthesis of fibrinogen as an acute phase protein in COVID-19 [66].

#### Impaired diffusion capacity

Lung diffusion capacity (DLCO) can be impaired, although pure diffusion defects are rarely a cause for increased P(A-a)O_2_ gradient at rest [67, 68]. SARS-CoV-2 propagates within alveolar type II cells, where a large number of viral particles will be produced and released, followed by immune response mediated destruction of infected cells (virus-linked pyroptosis) [54]. Loss of alveolar epithelial cells and a pro-coagulant state cause the denuded basement membrane to be covered with debris, consisting of fibrin, dead cells, and complement activation products, collectively referred to as hyaline membranes [54, 69]. With incremental exercise and in the face of absent hypoxic vasoconstriction in COVID-19, a hyperdynamic pulmonary circulation might not allow sufficient time for red blood cells to equilibrate their oxygen uptake. A diffusion limitation may, therefore, occur in COVID-19 leading to a raised P(A-a)O_2_ gradient and exercise-induced arterial hypoxemia (EIAH) [68]. Recently, Xiaoneng Mo et al. confirmed a decrease in DLCO in COVID-19 patients at the time of discharge. The prevalence of impaired diffusing-capacity was linked to the severity of disease, respectively 30.4% in mild illness, 42.4% in pneumonia and 84.2% in severe pneumonia [70]. Long-term studies are needed to address whether these deficits are persistent as seen in MERS where 37% of MERS survivors still presented with an impairment of DLCO [39].

### Preservation of lung mechanics

The outline presented in the previous paragraphs largely clarifies the dissociation between the severity of hypoxemia in COVID-19 and relatively well-preserved lung mechanics. Gas exchange abnormalities in some patients with COVID-19 occur earlier than increases in mechanical loads [41]. During the first days of infection, there is no increased airway resistance, and there is presumably no increased anatomical or physiological dead space ventilation. The breathing effort also remains rather low because lung compliance is normal in many patients without pre-existing lung disease. As recently shown by Gattinoni et al. in a cohort of 16 critically ill patients, relatively normal values for respiratory system compliance (50.2 ± 14.3 ml/cmH2O) went hand in hand with a dramatically increased shunt fraction of 0.50 ± 0.11 [47]. Such a wide discrepancy is highly unusual for most forms of disorders that lead to acute lung injury and ARDS [47, 71]. Relatively high compliance indicates a well-preserved lung gas volume and explains in part the absence of dyspnea early in the course of illness [42, 47, 61, 72, 73]. In contrast, Ziehr et al. described a low compliance and a uniform presentation consistent with the Berlin definition for ARDS in a cohort of COVID-19 patients [30, 70]. Of note, patients on mechanical ventilation have the highest COVID-19 severity and thus probably the lowest respiratory system compliance. Dyspnea itself may have precipitated mechanical ventilation, and the latter may be a surrogate marker for low compliance in COVID-19 [41]. Understanding of the respiratory mechanics found in COVID-19 will continue to evolve as further research is reported.

### Rapid deterioration

Hypoxemia-driven tachypnoea, hyperpnea and altered oxygenation predict clinical deterioration induced by either disease severity and/or host response and/or suboptimal management [35, 61]. As the disease progresses, the more consolidated air spaces do not inflate as easily at higher transpulmonary pressures. The volume loss is proportionally greater at higher lung volumes. This loss of volume reduces total lung compliance and increases the work of breathing [45]. There is also evidence that the dynamic compliance of the remaining ventilated lung is reduced in SARS-CoV-2 pneumonia (as seen in pneumococcal pneumonia) most possibly by a reduction in surfactant activity, further increasing the work of breathing [45]. Physiological dead space is also increasing due to reduced blood flow caused by intravascular thrombi. Importantly, the anxiety experienced by COVID-19 patients also affects the cortical feedback to the respiratory centers. Consequently, as the disease progresses, dyspnea becomes increasingly apparent.

## Thoughts on management

At the stage that COVID-19 patients are admitted to the hospital with hypoxemia, viral replication is well underway and in addition to giving antiviral medication, optimization of the V/Q mismatch and reduction of cytokine storm remain the major therapeutic goals. Regarding perfusion, avoiding microthrombi and ongoing fibrin deposition is one of the therapeutic strategies. It seems prudent to use thromboprophylaxis in all COVID-19 patients, particularly in those with high D-dimers on admission [59, 66, 74]. Moore et al. recently suggested the use of tissue plasminogen activator (tPA) to treat ARDS in COVID-19 [75]. In addition, tackling the systemic prothrombotic complication using anti-inflammatory medications (such as anti-IL6R tocilizumab or sarilumab, or the anti-IL6 antibody siltuximab or complement inhibiting strategies) to prevent macro- and microthrombi represents another potential approach and several trials are currently verifying this hypothesis [54]. Effective hypoxic pulmonary vasoconstriction may be another target to improve the matching of regional perfusion and ventilation in the lung. There is an excessive release of inflammatory mediators that disturbs the balance between nitric oxide (NO), endothelin, and prostanoids in the pulmonary capillaries [76], although inhaled NO has consistently failed to show an improvement in mortality in ARDS [53–55, 84]. RAS modulation (e.g. angiotensin receptor blockers, recombinant soluble ACE2, and inhibition of the bradykinin system) may have a potential role in restoring lung perfusion regulation, and trials are ongoing [50–52].

Regarding ventilation, supplemental oxygen is the first step in facilitating oxygenation. In patients with refractory hypoxemic respiratory failure (increasing shunt fraction), timely but not premature intubation and invasive ventilation support may be superior to non-invasive ventilation in boosting transpulmonary pressure, opening collapsed alveoli, improving oxygenation, decreasing oxygen debt, avoiding P-SILI and offering a better chance for the lungs to heal [61, 76, 77]. Considering the critical cases, most patients fulfil the Berlin criteria of ARDS, where lung-protective ventilation, prone ventilation, effective sedation and analgesia, and high positive end-expiratory pressure (PEEP) are key [54, 61, 70, 77]. COVID-19 patients are exquisitely PEEP sensitive [78–80]. Tolerance for modest permissive hypercapnia minimizes ventilator-induced lung injury (VILI) [61]. Since prone position recruits the dorsal lung regions and diverts blood flow to these caudal regions, it may have particular importance in COVID-19 when used early and in relatively long sessions [42]. Although further trials are needed to evaluate the impact on disease severity and mortality, several authors confirmed that awake proning can improve oxygenation in COVID-19 [81–83].

## Conclusion

The remarkable dissociation between profound hypoxemic respiratory failure and a clinically ‘happy’ patient is frequently seen and should prompt physicians and health care workers not only to rely on the patient’s apparent wellbeing but closely monitor respiratory rate, signs of hyperventilation, oxygen saturation and invasive measurements of hypoxemia/hypocapnia at regular time intervals. Pulse oximetry should be interpreted with caution, because left-sided shifting of the oxyhemoglobin dissociation curve. The arterial hypoxemia is induced by intrapulmonary shunting, dysregulated hypoxic pulmonary vasoconstriction, impaired lung diffusion, and formation of intravascular microthrombi. As in the first days of the disease, the lung mechanics are well-preserved and there is no increased airway resistance or dead space ventilation. The respiratory center thus does not sense an uncomfortable sensation of breathing. However, sudden and rapid respiratory decompensation may occur, and tachypnea and hyperpnea might be the most important clinical warning signs of impending respiratory failure in COVID-19 patients.

## Data Availability

Not applicable.

## Abbreviations

COVID-19: Coronavirus Disease 2019
SARS-CoV-2: Severe Acute Respiratory Syndrome Coronavirus 2
MERS: Middle East Respiratory Syndrome
ICU: Intensive Care Unit
PaCO_2_: Partial gas pressure of dissolved carbon dioxide in the blood
PaO_2_: Partial gas pressure of dissolved oxygen in the blood
SpO_2_: Oxygen saturation measured by pulse oximetry
P(A-a)O_2_ gradient: the alveolar to arterial oxygen gradient
VCO2: Rate of CO2 production
VE: Minute ventilation
VD: Dead space
Vt: Tidal volume
V/Q: Ventilation/Perfusion
F_i_O_2_: Fraction of inspired oxygen
P-SILI: Patient self-inflicted lung injury
VILI: Ventilator-induced lung injury
RAS: Renin-angiotensin system
ACE2: Angiotensin-converting enzyme 2
Ang: Angiotensin
TMA: Thrombotic microangiopathy
PAI: Plasminogen activator inhibitor
DIC: Diffuse intravascular coagulation
DLCO: Lung diffusion capacity
NO: Nitric oxide
PEEP: Positive end-expiratory pressure

## References

[1] Wu Z, McGoogan JM (2020). Characteristics of and important lessons from the coronavirus disease 2019 (COVID-19) outbreak in China: summary of a report of 72314 cases from the Chinese Center for Disease Control and Prevention. JAMA.

[2] Omer SB, Malani P, Del Rio C. The COVID-19 pandemic in the US: a clinical update. JAMA. 2020. Epub ahead of print. 10.1001/jama.2020.5788 32250388

[3] Verity R, Okell LC, Dorigatti I, Winskill P, Whittaker C, Imai N, et al. Articles estimates of the severity of coronavirus disease 2019: a model-based analysis. Lancet Infect Dis. 2020; [cited 2020 Apr 30]; Available from: www.thelancet.com/infection. 10.1016/S1473-3099(20)30243-7 PMC7158570 32240634

[4] Xie J, Tong Z, Guan X, Du B, Qiu H, Slutsky AS (2020). Critical care crisis and some recommendations during the COVID-19 epidemic in China. Intensive Care Med.

[5] Tobin MJ, Laghi F, Jubran A. Why COVID-19 silent hypoxemia is baffling to physicians. Am J Respir Crit Care Med. 2020; [cited 2020 Jun 23]; Available from: http://www.ncbi.nlm.nih.gov/pubmed/32539537. 10.1164/rccm.202006-2157CP PMC7397783 32539537

[6] Couzin-Frankel J. The mystery of the pandemic’s ‘happy hypoxia’ [internet]. Science (80- ). 2020:455–6 [cited 2020 Jun 23]. American Association for the Advancement of Science; Available from: https://science.sciencemag.org/content/368/6490/455. 10.1126/science.368.6490.455 32355007

[7] Wilkerson RG, Adler JD, Shah NG, Brown R. Silent hypoxia: a harbinger of clinical deterioration in patients with COVID-19. Am J Emerg Med. 2020; W.B. Saunders; [cited 2020 Jun 23]; Available from: https://pubmed.ncbi.nlm.nih.gov/32471783/. 10.1016/j.ajem.2020.05.044 PMC7243756 32471783

[8] Allali G, Marti C, Grosgurin O, Morélot-Panzini C, Similowski T, Adler D. Dyspnea: the vanished warning symptom of COVID-19 pneumonia. J Med Virol. 2020;jmv.26172 Wiley; [cited 2020 Jul 4]. Available from: https://onlinelibrary.wiley.com/doi/abs/10.1002/jmv.26172. 10.1002/jmv.26172 PMC7307122 32530534

[9] Xie J, Covassin N, Fan Z, Singh P, Gao W, Li G, et al. Association between hypoxemia and mortality in patients with COVID-19. Mayo Clin Proc. 2020; Elsevier; [cited 2020 Apr 19];0. Available from: https://linkinghub.elsevier.com/retrieve/pii/S0025619620303670. 10.1016/j.mayocp.2020.04.006 PMC7151468 32376101

[10] Siddiqi HK, Mehra MR. COVID-19 illness in native and immunosuppressed states: a clinical-therapeutic staging proposal. J Heart Lung Transplant. Elsevier BV. 2020;0. Epub ahead of print. 10.1016/j.healun.2020.03.012 PMC7118652 32362390

[11] Vaporidi K, Akoumianaki E, Telias I, Goligher EC, Brochard L, Georgopoulos D (2020). Respiratory drive in critically ill patients pathophysiology and clinical implications. Am J Respir Crit Care Med.

[12] Epstein FH, Manning HL, Schwartzstein RM. Pathophysiology of dyspnea [internet]. Epstein FH, editor. N Engl J Med. 1995:1547–53 Massachusetts Medical Society ; [cited 2020 Apr 19]. Available from: http://www.nejm.org/doi/10.1056/NEJM199512073332307. 10.1056/NEJM199512073332307 7477171

[13] Costa R, Navalesi P, Cammarota G, Longhini F, Spinazzola G, Cipriani F (2017). Remifentanil effects on respiratory drive and timing during pressure support ventilation and neurally adjusted ventilatory assist. Respir Physiol Neurobiol.

[14] Corne S, Webster K, C Ginn GM, St-john W, Younes M. Medullary metastasis causing impairment of respiratory pressure output with intact respiratory rhythm. Am J Respir Crit Care Med. 1999; Available from: www.atsjournals.org. 10.1164/ajrccm.159.1.9803051 9872856

[15] Richards JB (2017). Calculated decisions: mMRC (modified Medical Research Council) dyspnea scale. Emerg Med Pract.

[16] Fletcher CM, Elmes PC, Fairbairn AS, Wood CH (1959). The significance of respiratory symptoms and the diagnosis of chronic bronchitis in a working population. Br Med J.

[17] Raux M, Ray P, Prella M, Duguet A, Demoule A, Similowski T (2007). Cerebral cortex activation during experimentally induced ventilator fighting in normal humans receiving noninvasive mechanical ventilation. Anesthesiology.

[18] Esnault P, Roubin J, Cardinale M, D’Aranda E, Montcriol A, Cungi PJ (2019). Spontaneous hyperventilation in severe traumatic brain injury: incidence and association with poor neurological outcome. Neurocrit Care.

[19] Hamilton RD, Winning AJ, Perry A, Guz A (1987). Aerosol anesthesia increases hypercapnic ventilation and breathlessness in laryngectomized humans. J Appl Physiol.

[20] Manning HL, Shea SA, Schwartzstein RM, Lansing RW, Brown R, Banzett RB (1992). Reduced tidal volume increases “air hunger” at fixed PCO2 in ventilated quadriplegics. Respir Physiol.

[21] Dempsey JA, Smith CA. Pathophysiology of human ventilatory control: number 6 in the series “physiology in respiratory medicine”. Eur Respir JEuropean Respiratory Society. 2014:495–512. Epub ahead of print. 10.1183/09031936.00048514 PMC4578297 24925922

[22] Leusen IR (1954). Chemosensitivity of the respiratory center; influence of CO2 in the cerebral ventricles on respiration. Am J Physiol.

[23] Guyenet PG, Bayliss DA. Neural control of breathing and CO2 homeostasis. NeuronCell Press. 2015:946–61. Epub ahead of print. 10.1016/j.neuron.2015.08.001 PMC4559867 26335642

[24] Kvale PA, Conway WA, Coates EO (1980). Continuous or nocturnal oxygen therapy in hypoxemic chronic obstructive lung disease. A clinical trial. Ann Intern Med.

[25] Gandevia SC, Killian K, McKenzie DK, Crawford M, Allen GM, Gorman RB (1993). Respiratory sensations, cardiovascular control, kinaesthesia and transcranial stimulation during paralysis in humans. J Physiol.

[26] Easton PA, Slykerman LJ, Anthonisen NR (1986). Ventilatory response to sustained hypoxia in normal adults. J Appl Physiol.

[27] Gattinoni L, Chiumello D, Caironi P, Busana M, Romitti F, Brazzi L, et al. COVID-19 pneumonia : different respiratory treatment for different phenotypes ? Intensive Care Med. 2020:1–6 Springer; [cited 2020 Apr 19]. Available from: https://www.esicm.org/wp-content/uploads/2020/04/684_author-proof.pdf. 10.1007/s00134-020-06033-2 PMC7154064 32291463

[28] Mchenry LC, Slocum HC, Bivens HE, Mayes HA, Hayes GJ (1965). Hyperventilation in awake and anesthetized man: effects on cerebral blood flow and cerebral metabolism. Arch Neurol.

[29] Meng L, Gelb AW. Regulation of cerebral autoregulation by carbon dioxide. Anesthesiology. 2015:196–205 Lippincott Williams and Wilkins; [cited 2020 Jun 23]. Available from: https://pubmed.ncbi.nlm.nih.gov/25401418/. 10.1097/ALN.0000000000000506 25401418

[30] Wilkerson RG, Adler JD, Shah NG, Brown R. Silent hypoxia: a harbinger of clinical deterioration in patients with COVID-19. Am J Emerg Med. 2020; W.B. Saunders; [cited 2020 May 31]; Available from: https://linkinghub.elsevier.com/retrieve/pii/S0735675720303909. 10.1016/j.ajem.2020.05.044 PMC7243756 32471783

[31] Guan W, Ni Z, Hu Y, Liang W, Ou C, He J, et al. Clinical characteristics of coronavirus disease 2019 in China. N Engl J MedMassachusetts Medical Society. 2020. Epub ahead of print. 10.1056/NEJMoa2002032 PMC7092819 32109013

[32] D’Alonzo GE, Dantzker DR (1983). Respiratory failure, mechanisms of abnormal gas exchange, and oxygen delivery. Med Clin North Am.

[33] Ottestad W, Søvik S. COVID-19 patients with respiratory failure: what can we learn from aviation medicine? Br J AnaesthElsevier BV. 2020. Epub ahead of print. 10.1016/j.bja.2020.04.012 PMC7165289 32362340

[34] Hamilton C, Steinlechner B, Gruber E, Simon P, Wollenek G (2004). The oxygen dissociation curve: quantifying the shift. Perfusion.

[35] Woyke S, Rauch S, Ströhle M, Gatterer H. Modulation of Hb-O2 affinity to improve hypoxemia in COVID-19 patients. Clin NutrChurchill Livingstone. 2020. Epub ahead of print. 10.1016/j.clnu.2020.04.036 PMC7195129 32360083

[36] Sainburg RL, Clark AL, Billman GE, Schlader ZJ, Mündel T, Milne K, et al. Hypoxia, focus hypobaric hypoxia. Encycl Exerc Med Heal DisSpringer Berlin Heidelberg. 2012:428–31. Epub ahead of print.

[37] Liu W, Hualan L. COVID-19:attacks the 1-Beta chain of hemoglobin and captures the porphyrin to inhibit human Heme metabolism. ChemRxiv. 2020. Epub ahead of print.

[38] A-a O_2_ Gradient - MDCalc [Internet]. [cited 2020 Apr 30]. Available from: https://www.mdcalc.com/a-a-o2-gradient.

[39] Park WB, Il JK, Kim G, Choi JP, Rhee JY, Cheon S, et al. Correlation between pneumonia severity and pulmonary complications in Middle East respiratory syndrome. J Korean Med SciKorean Academy of Medical Science. 2018:33. Epub ahead of print. 10.3346/jkms.2018.33.e169 PMC5990444 29892209

[40] Barach AL, Martin J, Eckman M (1938). Positive pressure respiration and its application to the treatment of acute pulmonary edema. Ann Intern Med.

[41] Komorowski M, Aberegg SK. Using applied lung physiology to understand COVID-19 patterns. Br J Anaesth. 2020;0 Elsevier; [cited 2020 May 31]. Available from: https://linkinghub.elsevier.com/retrieve/pii/S0007091220303755. 10.1016/j.bja.2020.05.019 PMC7250770 32536444

[42] Archer SL, Sharp WW, Weir EK. Differentiating COVID-19 pneumonia from acute respiratory distress syndrome (ARDS) and high altitude pulmonary edema (HAPE): therapeutic implications. CirculationOvid Technologies (Wolters Kluwer Health). 2020. Epub ahead of print. 10.1161/CIRCULATIONAHA.120.047915 PMC7363563 32369390

[43] Lang M, Som A, Mendoza DP, Flores EJ, Reid N, Carey D, et al. Hypoxaemia related to COVID-19: vascular and perfusion abnormalities on dual-energy CT. Lancet Infect DisLancet Publishing Group. 2020. Epub ahead of print. 10.1016/S1473-3099(20)30367-4 PMC7252023 32359410

[44] Nagaraj C, Tabeling C, Nagy BM, Jain PP, Marsh LM, Papp R, et al. Hypoxic vascular response and ventilation/perfusion matching in end-stage COPD may depend on p22phox. Eur Respir JEuropean Respiratory Society. 2017;50. Epub ahead of print. 10.1183/13993003.01651-2016 28729471

[45] Light RB. Pulmonary pathophysiology of pneumococcal pneumonia. Semin Respir Infect. 1999:218–26. Epub ahead of print. 10501309

[46] van Heerden PV, Cameron PD, Karanovic A, Goodman MA (2003). Orthodeoxia - an uncommon presentation following bilateral thoracic sympathectomy. Anaesth intensive care. SAGE PublicationsSage UK: London. England.

[47] Gattinoni L, Coppola S, Cressoni M, Busana M, Chiumello D. Covid-19 does not Lead to a “typical” acute respiratory distress syndrome. Am J Respir Crit Care MedAmerican Thoracic Society. 2020. Epub ahead of print. 10.1164/rccm.202003-0817LE PMC7233352 32228035

[48] Cheng H, Wang Y, Wang GQ. Organ-protective effect of angiotensin-converting enzyme 2 and its effect on the prognosis of COVID-19. J Med VirolJohn Wiley and Sons Inc. 2020. Epub ahead of print. 10.1002/jmv.25785 PMC7317908 32221983

[49] South AM, Diz D, Chappell MC. COVID-19, ACE2 and the cardiovascular consequences. Am J Physiol Heart Circ PhysiolNLM (Medline). 2020. Epub ahead of print. 10.1152/ajpheart.00217.2020 PMC7191628 32228252

[50] Kickbusch I, Leung G. Response to the emerging novel coronavirus outbreak. BMJBMJ Publishing Group. 2020. Epub ahead of print. 10.1136/bmj.m406 32005675

[51] Zhang H, Baker A. Recombinant human ACE2: acing out angiotensin II in ARDS therapy. Crit Care. 2017:305 BioMed Central Ltd. [cited 2020 Apr 28]. Available from: https://ccforum.biomedcentral.com/articles/10.1186/s13054-017-1882-z. 10.1186/s13054-017-1882-z PMC5729230 29237475

[52] Vaduganathan M, Vardeny O, Michel T, McMurray JJV, Pfeffer MA, Solomon SD. Renin–angiotensin–aldosterone system inhibitors in patients with Covid-19. N Engl J MedMassachusetts Medical Society. 2020. Epub ahead of print. 10.1056/NEJMsr2005760 PMC7121452 32227760

[53] Wang S, Guo F, Liu K, Wang H, Rao S, Yang P (2008). Endocytosis of the receptor-binding domain of SARS-CoV spike protein together with virus receptor ACE2. Virus Res.

[54] Tay MZ, Poh CM, Rénia L, MacAry PA, Ng LFP. The trinity of COVID-19: immunity, inflammation and intervention. Nat Rev ImmunolNature Research. 2020:363–74. Epub ahead of print. 10.1038/s41577-020-0311-8 PMC7187672 32346093

[55] Liu Y, Yang Y, Zhang C, Huang F, Wang F, Yuan J (2020). Clinical and biochemical indexes from 2019-nCoV infected patients linked to viral loads and lung injury. Sci China Life Sci.

[56] Jia HP, Look DC, Shi L, Hickey M, Pewe L, Netland J (2005). ACE2 receptor expression and severe acute respiratory syndrome coronavirus infection depend on differentiation of human airway epithelia. J Virol.

[57] Bikdeli B, Madhavan MV, Jimenez D, Chuich T, Dreyfus I, Driggin E, et al. COVID-19 and thrombotic or thromboembolic disease: implications for prevention, antithrombotic therapy, and follow-up. J Am Coll Cardiol. 2020; Journal of the American College of Cardiology; [cited 2020 Apr 20]; Available from: https://linkinghub.elsevier.com/retrieve/pii/S0735109720350087. 10.1016/j.jacc.2020.04.031 PMC7164881 32311448

[58] Guérin C, Matthay MA. Acute cor pulmonale and the acute respiratory distress syndrome. Intensive Care MedSpringer Verlag. 2016:934–6. Epub ahead of print. 10.1007/s00134-015-4197-z 26759013

[59] Campbell CM, Kahwash R. Will complement inhibition be the new target in treating COVID-19 related systemic thrombosis? [cited 2020 Apr 20]; Available from: http://ahajournals.org. 10.1161/CIRCULATIONAHA.120.047419 32271624

[60] Zhang L, Yan X, Fan Q, Liu H, Liu X, Liu Z, et al. D-dimer levels on admission to predict in-hospital mortality in patients with Covid-19. J Thromb Haemost. 2020; [cited 2020 Apr 25]; Available from: http://www.ncbi.nlm.nih.gov/pubmed/32306492. 10.1111/jth.14859 PMC7264730 32306492

[61] Marini JJ, Gattinoni L. Management of COVID-19 respiratory distress. JAMA. 2020; [cited 2020 Apr 26]; Available from: https://jamanetwork.com/journals/jama/fullarticle/2765302. 10.1001/jama.2020.6825 32329799

[62] Wang J, Hajizadeh N, Moore EE, McIntyre RC, Moore PK, Veress LA, et al. Tissue plasminogen activator (tPA) treatment for COVID-19 associated acute respiratory distress syndrome (ARDS): a case series. J Thromb HaemostNLM (Medline). 2020. Epub ahead of print. 10.1111/jth.14828 PMC7262152 32267998

[63] Monsalve-Naharro JÁ, Domingo-Chiva E, Castillo SG, Cuesta-Montero P, Jiménez-Vizuete JM. Inhaled nitric oxide in adult patients with acute respiratory distress syndrome. Farm HospSociedad Espanola de Farmacia Hospitalaria. 2017:292–312. Epub ahead of print. 10.7399/fh.2017.41.2.10533 28236803

[64] Luo W, Yu H, Gou J, Li X, Sun Y, Li J (2020). Title: clinical pathology of critical patient with novel coronavirus pneumonia (COVID-19) list of authors.

[65] Tian S, Hu W, Niu L, Liu H, Xu H, Xiao S-Y (2020). Pulmonary pathology of early-phase 2019 novel coronavirus (COVID-19) pneumonia in two patients with lung cancer. J Thorac Oncol.

[66] Whyte CS, Morrow GB, Mitchell JL, Chowdary P, Mutch NJ. Fibrinolytic abnormalities in acute respiratory distress syndrome (ARDS) and versatility of thrombolytic drugs to treat COVID-19. J Thromb Haemost. 2020;jth.14872 [cited 2020 Apr 25]. Available from: https://onlinelibrary.wiley.com/doi/abs/10.1111/jth.14872. 10.1111/jth.14872 PMC7264738 32329246

[67] Di Marco F, Devaquet J, Lyazidi A, Galia F, Da Costa NP, Fumagalli R (2010). Positive end-expiratory pressure-induced functional recruitment in patients with acute respiratory distress syndrome. Crit Care Med.

[68] Hopkins SR. Exercise induced arterial hypoxemia: the role of ventilation-perfusion inequality and pulmonary diffusion limitation. Adv Exp Med BiolSpringer New York. 2006:17–30. Epub ahead of print. 10.1007/978-0-387-34817-9_3 17089876

[69] Mason RJ. Pathogenesis of COVID-19 from a cell biologic perspective. Eur Respir J. 2020; European Respiratory Society; [cited 2020 Apr 20]; Available from: http://www.ncbi.nlm.nih.gov/pubmed/32269085. 10.1183/13993003.00607-2020 PMC7144260 32269085

[70] Ziehr DR, Alladina J, Petri CR, Maley JH, Moskowitz A, Medoff BD, et al. Respiratory pathophysiology of mechanically ventilated patients with COVID-19: a cohort study. Am J Respir Crit Care MedNLM (Medline). 2020. Epub ahead of print. 10.1164/rccm.202004-1163LE PMC7301734 32348678

[71] Gattinoni L, Chiumello D, Rossi S (2020). COVID-19 pneumonia: ARDS or not?. Crit Care.

[72] Ottestad W, Seim M, Mæhlen JO. Covid-19 med stille hypoksemi. Tidsskr Nor LaegeforenNLM (Medline). 2020;140. Epub ahead of print. 10.4045/tidsskr.20.0299 32378842

[73] Marini JJ, Gattinoni L. Management of COVID-19 respiratory distress. JAMA - J Am Med AssocAmerican Medical Association. 2020. Epub ahead of print. 10.1001/jama.2020.6825 32329799

[74] Spyropoulos AC, Ageno W, Barnathan ES. Hospital-based use of thromboprophylaxis in patients with COVID-19. Lancet. 2020;0 Elsevier; [cited 2020 Apr 25]. Available from: https://linkinghub.elsevier.com/retrieve/pii/S0140673620309260. 10.1016/S0140-6736(20)30926-0 PMC7173816 32330428

[75] Wang J, Hajizadeh N, Moore EE, McIntyre RC, Moore PK, Veress LA, et al. Tissue plasminogen activator (tPA) treatment for COVID-19 associated acute respiratory distress syndrome (ARDS): a case series. J Thromb Haemost. 2020; [cited 2020 Apr 25]; Available from: http://www.ncbi.nlm.nih.gov/pubmed/32267998. 10.1111/jth.14828 PMC7262152 32267998

[76] Tarry D, Powell M (2017). Hypoxic pulmonary vasoconstriction. BJA Educ.

[77] Meng L, Qiu H, Wan L, Ai Y, Xue Z, Guo Q, et al. Intubation and ventilation amid the COVID-19 outbreak. AnesthesiologyOvid Technologies (Wolters Kluwer Health). 2020;1. Epub ahead of print.

[78] Dondorp AM, Hayat M, Aryal D, Beane A, Schultz MJ. Respiratory support in novel coronavirus disease (COVID-19) patients, with a focus on resource-limited settings. Am J Trop Med Hyg. 2020; [cited 2020 Apr 25]; Available from: http://www.ncbi.nlm.nih.gov/pubmed/32319424. 10.4269/ajtmh.20-0283 PMC7253105 32319424

[79] Guérin C, Reignier J, Richard J-C, Beuret P, Gacouin A, Boulain T (2013). Prone positioning in severe acute respiratory distress syndrome. N Engl J Med.

[80] Elharrar X, Trigui Y, Dols A-M, Touchon F, Martinez S, Prud’homme E, et al. Use of prone positioning in nonintubated patients with COVID-19 and hypoxemic acute respiratory failure. JAMAAmerican Medical Association (AMA). 2020. Epub ahead of print. 10.1001/jama.2020.8255 PMC7229532 32412581

[81] Caputo ND, Strayer RJ, Levitan R (2020). Early self-Proning in awake, non-intubated patients in the emergency department: a single ED’s experience during the COVID-19 pandemic. Kline J, editor. Acad Emerg Med.

[82] Ding L, Wang L, Ma W, He H. Efficacy and safety of early prone positioning combined with HFNC or NIV in moderate to severe ARDS: a multi-center prospective cohort study. Crit CareBioMed Central Ltd. 2020;24. Epub ahead of print. 10.1186/s13054-020-2738-5 PMC6993481 32000806

[83] Slessarev M, Cheng J, Ondrejicka M, Arntfield R. Patient self-proning with high-flow nasal cannula improves oxygenation in COVID-19 pneumonia. Can J AnesthSpringer. 2020:1. Epub ahead of print. 10.1007/s12630-020-01661-0 PMC7172385 32319029

[84] Monsalve-Naharro JÁ, Domingo-Chiva E, Castillo SG, Cuesta-Montero P, Jiménez-Vizuete JM. Inhaled nitric oxide in adult patients with acute respiratory distress syndrome. Farm Hosp. 2017:292–312 Sociedad Espanola de Farmacia Hospitalaria; [cited 2020 Apr 25]. Available from: http://www.ncbi.nlm.nih.gov/pubmed/28236803. 10.7399/fh.2017.41.2.10533 28236803

